# Central line–associated bloodstream infections and complications in adult home parenteral nutrition: Observations from a quality improvement initiative

**DOI:** 10.1002/ncp.11338

**Published:** 2025-06-29

**Authors:** Theresa A. Fessler, Mary B. Crandall, Sarah E. Schumacher, David N. Martin

**Affiliations:** ^1^ University of Virginia Health System Charlottesville Virginia USA; ^2^ Continuum Home Health, University of Virginia Health System Charlottesville Virginia USA; ^3^ Department of Nutrition Services, Morrison Healthcare University of Virginia Health System Charlottesville Virginia USA

**Keywords:** administration, adult, central line–associated bloodstream infection (CLABSI), home nutrition support, life cycle, nutrition, nutrition support practice, parenteral nutrition, venous access

## Abstract

The literature is inconsistent regarding differences in infection risk between central venous catheter types used for home parenteral nutrition (HPN). Our goal was to determine if significant differences exist in rates of infection and other complications between peripherally inserted central catheters, tunneled central venous catheters, and implanted ports, as well as between single and multiple lumen catheters used for HPN. Data were collected for 141 central venous catheters placed in 89 adults receiving HPN provided by Continuum home health company affiliated with University of Virginia health system. The catheters were 63% peripherally inserted, 27% tunneled, and 10% implanted ports, with 25,273 total catheter days and 15,474 HPN days. Central line–associated bloodstream infection (CLABSI) rates were 0.91 episodes per 1000 total catheter days overall, 1.91 for peripherally inserted, 0.63 for tunneled, and zero for ports. CLABSI rates per 1000 HPN days were 1.49 overall, 2.61 for peripherally inserted, and 0.76 for tunneled catheters. CLABSI rates were significantly greater for peripherally inserted than for tunneled catheters per total (*P* = 0.023) and per HPN (*P* = 0.011) catheter days. CLABSI rates were greater, but not significantly so, for multiple than for single lumen catheters. The rate of noninfectious complications was not significantly different between tunneled and peripherally inserted central catheters and was zero for ports. HPN CLABSI rates were significantly lower with implanted ports and tunneled central venous catheters as compared with peripherally inserted central catheters.

## INTRODUCTION

Parenteral nutrition (PN) is a life‐sustaining therapy administered via central venous access for patients experiencing gastrointestinal (GI) tract dysfunction. For individuals with long‐term or permanent GI dysfunction, home PN (HPN) allows for nutrition support in the home environment; however, this carries several risks. Catheter‐related infections are the most prevalent and serious complications for patients receiving HPN that escalate morbidity and healthcare costs. Risks vary with the type of central venous catheter (CVC), number of lumens, indication for HPN, type of medical insurance, and use of antimicrobial lock therapy, yet studies are not consistent on whether there are significant differences in infection risk related to catheter type.^1^ Definitions for infection tracking vary, with some researchers using central line–associated bloodstream infection (CLABSI) and some using catheter‐related bloodstream infection (CRBSI), as well as other differences in clinical and microbiological methods of diagnosis.^1^, ^2^, ^3^

According to the most recent American Society for Parenteral and Enteral Nutrition (ASPEN) Guidelines (2019), tunneled central venous catheters (TCVCs) should be used for adult patients anticipated to require long‐term PN, and if the duration is uncertain or expected to be <30 days, peripherally inserted central catheters (PICCs) can be used.^3^ Recent European Society for Clinical Nutrition and Metabolism (ESPEN) guidelines for HPN recommend TCVC or implanted ports for long‐term PN while acknowledging successful use of PICC lines for up to 4 years and recommending PICCs be used for PN of expected durations of <6 months.^4^ Despite these recommendations, current literature shows varied results regarding which type of CVC is safest to use for HPN in terms of risk for infection and other complications. At the time of this writing, there are no published randomized clinical trials comparing infection rates among the 3 different types of catheters: PICC, TCVC, and implanted ports.

In 2019, Hon et al reported significantly lower CRBSI risk with the use of PICC than TCVC using five comparative studies on adult HPN; however, in their meta‐analysis of 12 single‐arm studies, they found no difference in CRBSI risk between PICC and TCVC.^5^ Another meta‐analysis by Mateo‐Lobo et al examined three prospective observational HPN studies and found insufficient evidence of differences in CRBSI rates, thrombosis, or mechanical complications between PICC and TCVC.^6^, ^7^, ^8^, ^9^ In a systematic review and meta‐analysis of 53 studies, Reitzel et al noted incongruent results, as PICC lines were associated with increased CLABSI rates in some reports^10^, ^11^, ^12^ but decreased CRBSI^8^, ^9^ and decreased CLABSI rates in others.^1^, ^13^ Later, in 2020, Xue et al reported tunneled and implanted catheters to be risk factors for HPN CLABSI and less risk with PICC lines.^14^ Researchers found multiple lumen (ML) catheters are more likely to become infected than single lumen (SL) catheters used for HPN in four studies in the literature.^7^, ^13^, ^15^, ^16^ ASPEN recommends using the fewest number of lumens as feasible, and according to ESPEN guidelines, a single lumen is preferred to minimize infection risk.^3^, ^4^

Several studies have addressed noninfectious CVC complications for patients using HPN. The risk of thrombosis is low for TCVC and ports in the first year of use.^17^ One study found more thrombosis episodes with PICC as compared with none with TCVC.^9^ Another study found significantly more mechanical complications with PICC as compared with TCVC.^12^ In 2021, Matysiak et al reported no statistically significant differences in complication rates of tunneled SL PICC vs SL TCVC per 1000 catheter days.^18^ Cotogni et al found similar rates for mechanical complications and thrombosis for PICCs, TCVCs, and ports used for HPN in cancer patients.^19^

### Purpose

PICC lines are the most common CVC used for home PN in our health system. The purpose of this quality improvement (QI) project was to determine which type of central line presents the least risk of infection or other complications for our patients receiving HPN.

### Research questions

For patients receiving HPN from our hospital‐affiliated home health company, do significant differences exist in:

1) Incidence of CLABSI and CRBSI for PICC lines, TCVCs, and implanted ports?
2) Incidence of CLABSI for SL, double lumen (DL), or ML catheters?
3) Other noninfectious CVC‐related complications between PICC, TCVC, and port?
4) Are any clinical or demographic characteristics associated with a higher incidence of CLABSI?

## METHODS

We collected data on adult patients receiving HPN from University of Virginia Health System's Continuum Home Infusion company. This project was submitted for institutional review board review on June 18, 2019. After review, it did not meet the requirements of research with human subjects or clinical investigation and was determined to be a QI project, with approval to publish as such. The lead author discussed the project with the home health nursing management team and with the head infectious disease physicians at our hospital who agreed with the project being undertaken as an initial phase of a QI project to determine if there is a difference in infection rates or other complications between the three types of CVCs used for HPN.

### Cohort and data collection

The registered dietitian who followed adult patients receiving HPN for routine nutrition care collected information from the hospital electronic medical record (EMR) and our home care pharmacy EMR for all adult patients who were receiving service for HPN with our home infusion company as of February 2019 and through December 31, 2022. Data were collected prospectively from June 2019 and retrospectively to capture data from patients who were receiving service as of February 2019. Some patients in the cohort had CVCs that were placed months prior; with April 17, 2018, as the earliest HPN start date and March 22, 2018, as the earliest CVC (port) placement. Data collection included all CVCs used by each patient in the cohort to capture the full HPN experience of individual patients while receiving our service. As of January 1, 2023, no additional CVCs were added to the database. Follow‐up on CVCs still in use for HPN as of January 1, 2023, continued until July 31, 2023. One patient who reached age 18 years was transferred from the pediatric to the adult HPN service, and his information was added to the database in 2022. Some data were collected from outside hospital EMRs for patients whose CVCs were placed at other facilities or who were admitted to outside hospitals during the data collection period.

*Exclusions*: Of 96 patients, seven were excluded, leaving a total of 89 patients in the cohort. Exclusion reasons were numerous (>8) hospital readmissions for three patients; one patient with >6 hospital readmissions to an outside hospital with insufficient information; one patient for which we lacked sufficient information regarding CVC insertions and removals at an outside hospital; one patient who was noncompliant with HPN use and failed to communicate with the HPN team; and one patient with a <2‐day length of therapy. Pediatric patients and patients older than 18 years but still receiving service by the pediatric GI service were not included in the database.

Information recorded for the 141 central lines included patient age, gender, race, home nursing agency, diagnoses and indications for PN need, types of CVC used for HPN, dates of CVC insertion and removal, dates of HPN start and discontinuation, other CVC complications, reasons for CVC removal, and outcomes at the time of removal. Results of microbiologic blood tests from CVCs and peripheral vein blood draws, catheter tip cultures, identified microorganisms, and actual or potential alternate infection sources were recorded. Primary medical insurance was based on patients' coverage at the time of the CVC line insertion, and in a few cases, insurance payors changed during the observation period. The presence of fistula, ostomy, types of ostomies, and use of chemotherapy was also recorded. Clinical and demographic characteristics for the 89 patients and associated 141 CVCs are listed in Tables 1 and 2, respectively.

**Table 1:** Demographic and clinical characteristics of patients (*n* = 89).

Characteristics	Number of patients (*n* = 89)	Percentage	*P* value
Age (years) time of first CVC insertion			<0.001
17–29	8	9.0%	
30–45	24	27.0%	
46–56	29	32.6%	
57–64	17	19.1%	
66–73	8	9.0%	
76–78	3	3.4%	
Number of CVCs per patient			<0.001
1 line	37	41.6%	
2 lines	22	24.7%	
3 lines	13	14.6%	
4 lines	8	9.0%	
5 lines	4	4.5%	
6–9 lines	5	5.6%	
Male	36	40.4%	0.072
Female	53	59.6%	
Race			<0.001
Asian	2	2.2%	
Black	9	10.1%	
Hispanic	3	3.4%	
Other	2	2.2%	
White	73	82.0%	
Main or initial indication for parenteral nutrition			<0.001
Bowel obstruction	27	30.3%	
Short bowel	12	13.5%	
Leak or perforation	12	13.5%	
Enterocutaneous fistula	10	11.2%	
Nausea and vomiting after bariatric surgery	6	6.7%	
Malabsorption	3	3.4%	
Mesenteric ischemia	1	1.1%	
Ileus	1	1.1%	
Graft‐vs‐host disease	1	1.1%	
Other (refer to Figure 1)	16	18.0%	
Patients with no CLABSI	73	82.0%	
Patients with CLABSI^a^	16	18.0%	<0.001
Primary insurance at time of first CVC insertion			<0.001
Commercial	38	42.7%	
Medicare	25	28.1%	
Medicaid	23	25.8%	
Other	1	1.1%	
None	2	2.2%	
Chemotherapy during time CVC in place	11	12.4%	<0.001
No chemotherapy during time CVC in place	78	87.6%	
Cancer	42	47.2%	0.596
No cancer diagnosis	47	52.8%	
Ostomy types			<0.001
Colostomy	7	7.9%	
Ileostomy	16	18.0%	
Jejunostomy	1	1.1%	
Cecostomy	1	1.1%	
No ostomy	64	71.9%	
Fistula	18	20.2%	<0.001
No fistula	71	79.8%	
Fistula and ostomy^b^	9	10.1%	<0.001
No ostomy and no fistula	55	61.8%	
Fistula only	9	10.1%	
Ostomy only	16	18.0%	

Abbreviations: CLABSI, central line–associated bloodstream infection; CVC, central venous catheter.

^a^ Two patients had 2 CLABSIs, one patient had 3 CLABSIs, and one patient had 4 CLABSIs.

^b^ Eight with ileostomy, and one with colostomy.

**Table 2:** Clinical and demographic characteristics associated with CVCs used for HPN.

Characteristic	Number of CVCs (*n* = 141)	HPN CLABSI (*n* = 23)	HPN catheter days (*n* = 15,474)	HPN CLABSI rate per HPN catheter days	*P* value	Total catheter days (*n* = 25,273)	HPN CLABSI rate per total catheter days	*P* value
Male	59	8	6918	1.156	0.338	10,288	0.778	0.563
Female	82	15	8556	1.753		14,985	1.001	
Age <43 years	35	1	4109	0.243	**0.016**	7027	0.142	**0.012**
Age ≥43 years	106	22	11,365	1.936		18,246	1.206	
Home nursing	130	22	13,839	1.589	0.332	22,731	0.968	0.362
No home nursing	11	1	1635	0.612		2542	0.393	
No cancer	81	15	10,256	1.463	0.914	14,888	1.008	0.538
Cancer	60	8	5218	1.533		10,385	0.770	
Chemotherapy	12	1	606	1.650	0.915	1654	0.605	0.667
No chemotherapy	129	22	14,868	1.480		23,619	0.931	
Commercial insurance	42	3	5404	0.555	**0.003**	9465	0.317	**0.003**
Medicare	59	14	6983	2.005		10,271	1.363	
Medicaid	35	5	2794	1.790		5159	0.969	
No insurance	3	1	52	19.231		92	10.870	
Other	2	0	241	0		286	0	
Gastric bypass	13	1	392	2.551	0.579	759	1.318	0.705
No gastric bypass	128	22	15,082	1.459		24,514	0.897	
Asian	2	0	39	0	0.394	129	0	0.253
Black	12	2	846	2.364		1222	1.637	
Hispanic	3	1	207	4.831		247	4.049	
Other	2	0	1352	0		2022	0	
White	122	20	13,030	1.535		21,653	0.924	
Ostomy	40	7	5224	1.340	0.736	8300	0.843	0.806
No ostomy	101	16	10,250	1.561		16,973	0.943	
Fistula	32	6	1965	3.053	0.054	3624	1.656	0.108
No fistula	109	17	13,509	1.258		21,649	0.785	
Ostomy and fistula	19	6	1262	4.754	**0.023**	2132	2.814	**0.029**
No ostomy or fistula	88	16	9547	1.676		15,481	1.034	
Bowel obstruction	36	4	2899	1.380	0.171	7166	0.558	0.172
Short bowel	35	10	5929	1.687		7958	1.257	
Fistula	21	5	1652	3.027		2636	1.897	
GVHD	1	0	4	0		183	0	
Ileus	1	0	51	0		65	0	
Leak‐perforation	12	0	367	0		572	0	
Malabsorption	6	0	1702	0		2041	0	
Mesenteric ischemia	3	1	454	2.203		472	2.119	
N/V S/P bariatric surg	6	1	85	11.765		194	5.155	
Other	20	2	2331	0.858		3986	0.502	

Abbreviations: CLABSI, central line–associated bloodstream infection; CVC, central venous catheter; GVHD, graft versus host disease; HPN, home parenteral nutrition; N/V, nausea and vomiting; S/P, status post.

### Central venous access and CVC line care

Most CVCs in our cohort were placed by our hospital interventional radiology physicians, though a small number of lines were placed by providers from outside hospitals either prior to patients coming on service for home healthcare or while receiving care at their respective hospitals, prior to discharge home. Most central lines placed at our hospital were PICC: polyurethane Bard 4 French (F) SL PowerPICC and Bard 4 F DL PowerPICC. Most TCVCs were Bard 5 F SL PowerLine, Bard 6 F DL PowerLine, Medcomp 5 F SL Pro‐Line, and Medcomp 6 F DL Pro‐Line; and ports were Bard 8 F SL PowerPort M.R.I. Implantable port and Bard 8 F SL PowerPort ClearVUE (silicone).

The protocol for patient instruction included meeting with a home health liaison registered nurse for education prior to hospital discharge or in the home prior to the patients' first use of home PN. Patients were instructed to (1) cleanse a secure work area with antibacterial/antiseptic wipe or cleaner. (2) Wash hands, don gloves, and clean the CVC line hub for 15–30 s with alcohol wipes before performing any tasks with the CVC. (3) Unclamp the CVC, flush with normal saline solution using a method of 3 repetitions of “push‐pause,” then clean the hub with an alcohol wipe for at least 15 s prior to connecting the PN intravenous (IV) tubing to the CVC hub. (4) After completion of the PN infusion, flush the line with 5–10 ml of normal saline, then 3–5 ml (100 units/ml) of heparin, and lastly clean the hub again with an alcohol wipe for at least 15 s prior to clamping. One of the patients in the cohort who had a long‐term TCVC did not use heparin.

Patients were provided with a Medical Action Industries central line dressing kit, which included 1 Sorbaview 2000 (Vitality Medical) transparent dressing, 2 BD ChloraPrep Chlorhexidine applicators, 1 pair sterile nitrile gloves, 2 mask covers, drape, and tape. Clear CVC dressings were changed once weekly and more often if they became wet or soiled. CVC sites were cleaned with two chlorhexidine applicators during once‐weekly dressing changes. Patients with an allergy to chlorhexidine were provided with three iodine swabs, three alcohol swabs, and saline wipes to be used at time of the dressing change. Alternate dressings (for those with allergy or intolerance to our standard dressings) were either plain 3 M Tegaderm film or OP‐site IV 3000 by Smith and Nephew, measuring 10 × 12 cm per patients' preference. Use of chlorhexidine Biopatch was not part of our protocol; however, at least one patient in our cohort used it, as some patients with outside health system nursing agencies required it per their protocol. Although not part of our protocol, some patients used 3 M Curos 70% ethanol caps instead of standard caps to cover CVC hubs when not in use. “Green caps” were sometimes provided by hospital nurses before patients were discharged, and sometimes patients requested them from our home infusion company.

Patients' blood samples were drawn from their CVCs for routine testing, and none of our patients used taurolidine or ethanol locks during the data collection period.

### Our modified definitions of CLABSI and CRBSI

There is no standard CLABSI surveillance definition for home infusion patients. This makes it challenging to assess potential infections and accurately collect data for analysis. To standardize and validate our data collection for this project, we used a modified National Healthcare Safety Network (NHSN)–CLABSI definition to evaluate potential infection events. The Center for Disease Control and Prevention (CDC) NHSN has surveillance definitions used by hospitals and long‐term care facilities to identify and track healthcare‐associated infection. NHSN defines a CLABSI as a laboratory‐confirmed bloodstream infection (LCBI) in which an eligible bloodstream infection organism is identified and an eligible central line is present on the LCBI date of event or the day before.^20^

#### HPN CLABSI

− Bacteremia or fungemia in a patient receiving HPN with a CVC that was in use >2 calendar days before development of the bloodstream infection.
− A positive blood culture from a peripheral vein or central line in a patient using HPN that cannot be attributed to another infectious source, using NHSN definitions.
− If the organism is a common commensal, there must be a matching blood specimen that is collected on the same day or the next day and a clinical manifestation, such as fever (>38.0°C), chills, and/or hypotension.

#### HPN CRBSI estimate (Our modified criteria)

− Bacteremia or fungemia in a patient with a CVC that was in use >2 calendar days before the development of the bloodstream infection.

− Clinical symptoms of infection, such as fever, chills, malaise, or inflammation at the catheter insertion site.
− Positive blood cultures from both peripheral vein and central line, which are positive for the same organism.
− Infection is further verified by catheter tip culture, if available.
− The positive blood culture cannot be attributed to another infectious source, using NHSN definitions.

### Data analysis

Data were analyzed based on the number and type of CVCs. Infection and noninfectious complication rates were calculated in two ways: (1) by infections per 1000 total catheter days and (2) based on 1000 HPN catheter days. The primary investigator chose to calculate HPN catheter days to distinguish between time receiving HPN with our home infusion service as opposed to the days patients received HPN from other home infusion companies or during hospitalizations, thus omitting days when central lines were in place but not being used for HPN. CLABSIs occurring during hospitalizations on hospital day 3 or later were not counted as HPN CLABSI.

HPN CLABSI and other CVC complication rates were calculated by:

(numberofinfections÷totalcatheterdays)×1000

(numberofinfections÷HPNcatheterdays)×1000

(numberofnoninfectiouscomplications÷totalcatheterdays)×1000

(numberofnoninfectiouscomplications÷HPNcatheterdays)×1000

The number of total catheter days and HPN catheter days were calculated for each CVC. *Total catheter days* is the difference between the insertion date and removal date of the catheter or the end date of the observation period, whichever came first. If the removal date was unknown (15 CVCs), the last day of HPN was used, and if the insertion date was unknown (three CVCs), the first day of HPN was used to count total catheter days. HPN catheter days were defined as days that the patient was in the home setting and using PN and were calculated by the difference between the dates of HPN start and HPN discontinuation. For those who were readmitted to the hospital, hospitalized days were subtracted from the total HPN catheter days. A few patients traveled on occasion (such as vacation) or very rarely, were employed outside the home, and one patient traveled to another outside hospital facility clinic periodically for purposes of medication study. For purposes of data analysis, total and HPN catheter days were truncated to the end of the follow‐up period of July 31, 2023, for six patients who continued HPN after January 1, 2023.

CLABSI events were initially identified by positive blood cultures in combination with information from chart notes by infectious disease and attending physicians. The primary investigator identified 27 cases of potential CLABSI events for which CVCs were removed for infection. For nine of those cases of potential CLABSI, the infectious disease and/or attending physicians could not determine if the CVC was the source of infection; thus, we initially categorized these nine cases as “undetermined source.” In the second phase of this project, an infection preventionist (IP) expert categorized all potential infections by application of the modified NHSN criteria, as described earlier, to determine HPN CLABSI and to confirm infections originating from alternate sources in the body. CLABSI was ruled out in four of the original nine “undetermined source” cases. Our institution does not perform the specific microbiologic testing necessary to determine CRBSI; thus, we used a modified definition for CRBSI, with the intent to have an estimate of comparison with the published studies that reported CRBSI rates.

### Statistics

Data for this project were analyzed using *R* (4.4.0) and RStudio (2024.09.0). HPN infection rates were analyzed by the two‐sample *z* test of proportions and in some cases using a Bonferroni correction for multiple comparisons. Comparisons of CLABSI rates per total and HPN catheter days for the clinical and demographic variables were analyzed by the two‐proportions *z* test. In comparisons of more than two groups per variable, the *P* values refer to differences in at least one of the groups in contrast to another for that variable. Comparisons of noninfectious complications between PICC and TCVC were analyzed by the two‐sample *z* test of proportions. For the comparison of average age with and without CLABSI, *t* test was used, as age is a continuous variable. The chi‐squared goodness‐of‐fit test was used to compare the demographic and clinical characteristics of the 89 patients in Table 1.

## RESULTS

A total of 141 CVCs were used, as 52 of the 89 patients had more than one CVC placed for HPN during their course of therapy. The average age at time of initial central line insertion for the 89 patients was 49.4 years old (SD = 14 years). Of the 141 CVCs, 82 were placed in female patients (58%) and 59 were placed in male patients (42%.) Central line types were 63% PICC, 27% TCVC, and 10% implanted ports. Of the total 141 CVCs, 42.5% were SL, 55% DL, and 2% triple lumen. The total number of HPN catheter days was 15,474, whereas the total catheter days were 25,273. Of the 38 TCVCs, 28 (74%) were placed in the right internal jugular vein (IJ), eight in the left IJ, and two in the right subclavian vein. None of the patients used an ethanol lock during the observation period; however, two patients used ethanol lock in the past with their TCVCs before starting on our adult HPN service. See Table 2 for a list of clinical and demographic characteristics pertaining to the 141 CVCs.

**Figure 1:**
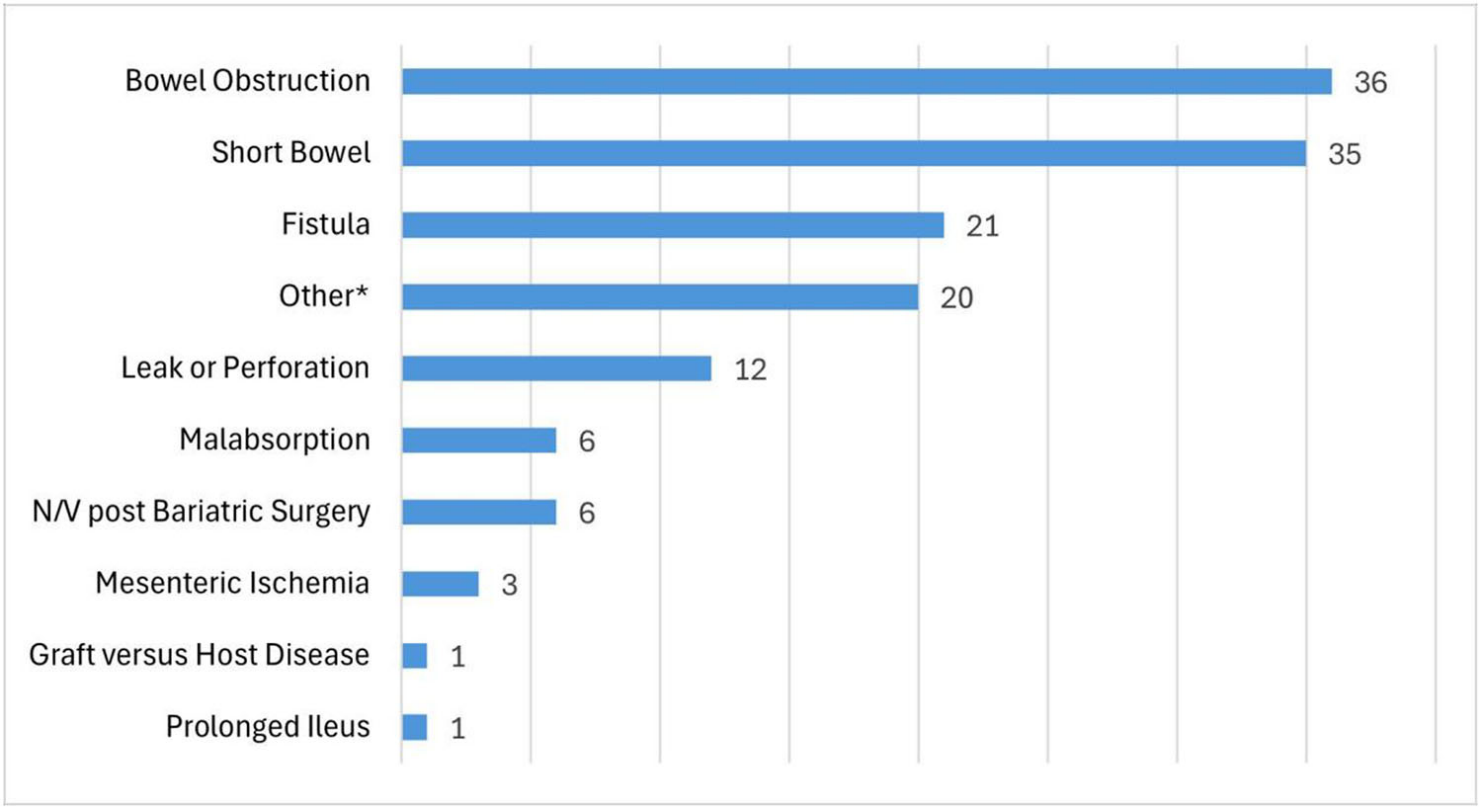
Indications for home parenteral nutrition for each central venous catheter (*n* = 141). N/V, nausea and/or vomiting. *Other includes intractable nausea and/or vomiting and/or inability to tolerate enteral feeding due to gastric outlet obstruction, unspecified dysmotility, superior mesenteric artery syndrome, pancreatitis, peritoneal carcinomatosis, high ileostomy output unresponsive to diet and medical therapy, and pseudoobstruction.

Of the total 141 CVCs, most indications for PN were for bowel obstruction (36), short bowel syndrome (35), and enterocutaneous fistula (21), as depicted in Figure 1. Other indications were leak or perforation (12), malabsorption (6), nausea and vomiting with intolerance of oral diet after bariatric surgery (6), mesenteric ischemia (3), ileus (1), graft‐vs‐host disease (1), and other indications (20). The “other” group included gastric outlet obstruction with intolerance of enteral feeding, unspecified dysmotility, superior mesenteric artery syndrome, pancreatitis with intolerance of enteral feeding, peritoneal carcinomatosis, high ileostomy output with failure of medical and diet therapy, and pseudoobstruction.

### Infection

**Figure 2:**
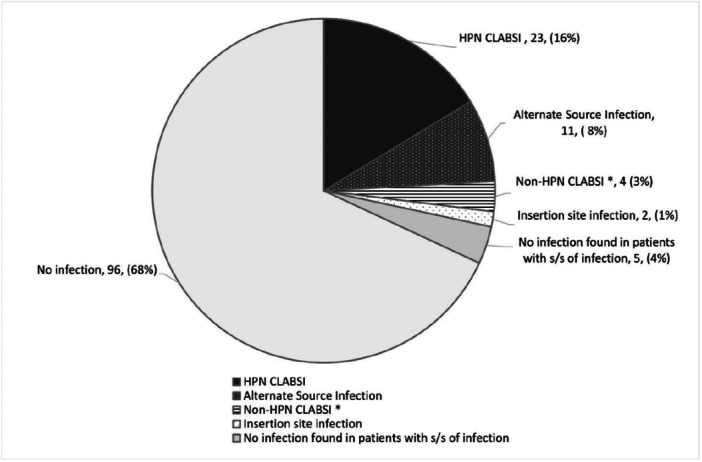
Status of 141 central venous catheters. CLABSI, central line–associated bloodstream infection; HPN, home parenteral nutrition; s/s, signs and symptoms. *Non‐HPN CLABSI: CLABSI that occurred while patients were in hospital or while not receiving HPN.

**Figure 3:**
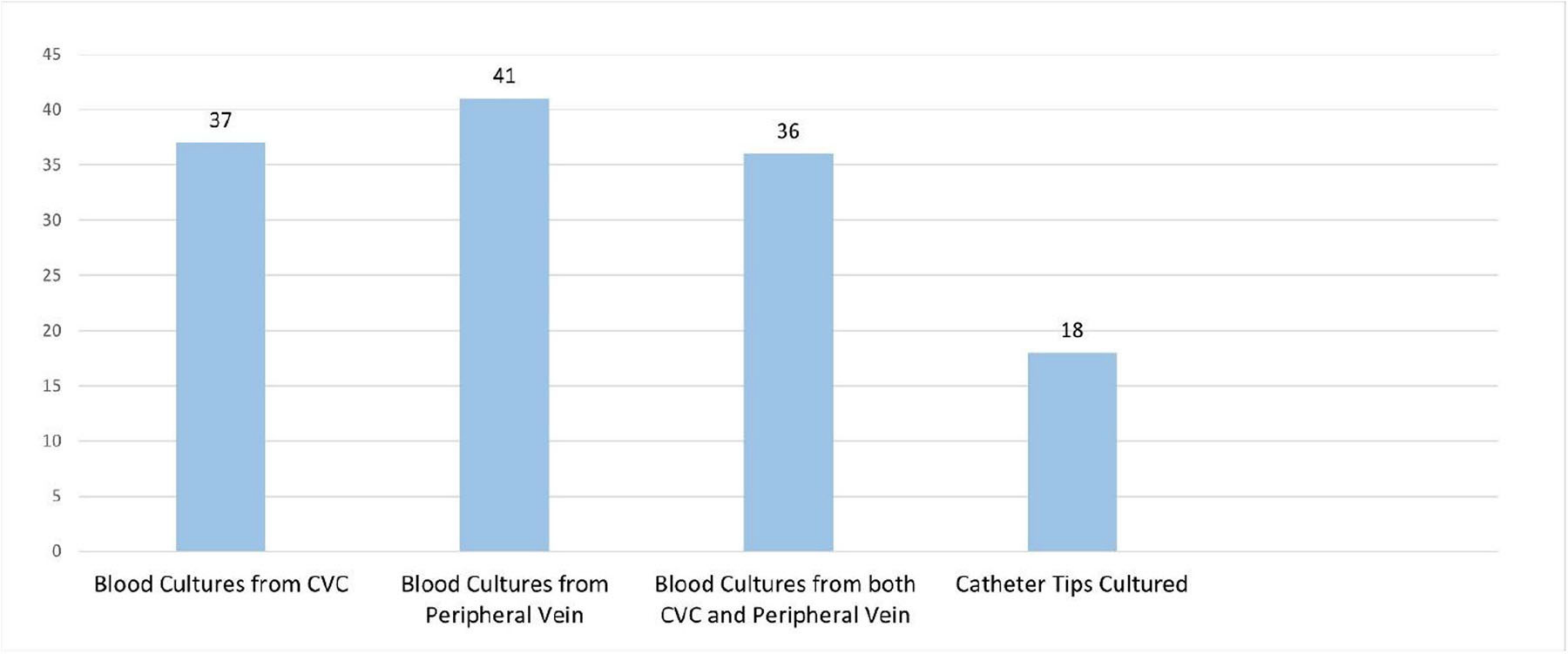
Tests done for 45 central venous catheters in patients with suspected infection (*n* = 45). CVC, central venous catheter.

Of the total 141 CVCs, 45 were in patients with signs or symptoms of infection at some point during their HPN therapy, as depicted in Figure 2. Of the 45 potential infection incidents, 23 met the criteria for HPN CLABSI, 13 of which also met our modified criteria to estimate CRBSI. Eleven of the 45 potential infection cases were found to be alternate‐source infections, four were cases of hospital or non‐HPN CLABSI, two were reported insertion‐site infections, and in five cases, no infection was found. As shown in Figure 3, 36 of the 45 (80%) potential infection cases had blood drawn from both a peripheral vein and the CVC and 18 catheter tips were cultured.

HPN CLABSI was confirmed in 23 cases, 17 with PICC lines, six with TCVCs, and none with ports, as shown in Table S1. CLABSI rate was 0.91 per 1000 total catheter days overall, with 1.91 for PICC, 0.63 for TCVC, and 0 for port, which is significantly higher for PICC lines (*P* = 0.023) as compared with TCVCs, as depicted in Table 3. When calculated per HPN catheter days, CLABSI rates were also significantly higher for PICC lines as compared with TCVCs, with 1.49 per HPN catheter days overall, 2.61 for PICC, and 0.76 for TCVC (*P* = 0.011). Table S2 shows total and HPN catheter days for the different catheter types. For the six TCVCs with CLABSI, five were in right IJ veins and one was in a right subclavian vein. One PICC CLABSI occurred in 2018 for an initial patient in the cohort.

**Table 3:** HPN CLABSI rates.

Infections	Overall (*n* = 23)	PICC (*n* = 17)	TCVC (*n* = 6)	*P* value*
CLABSI per 1000 total catheter days	0.91	1.91	0.63	0.023
CLABSI per 1000 HPN catheter days	1.49	2.61	0.76	0.011

*Note*: Port infections (*n* = 0); with port included in statistics using Bonferroni correction: port vs PICC (*P* = 0.00075) and port vs TCVC (*P* = 0.09838) for total catheter days; and port vs PICC (*P* = 0.17734) and port vs TCVC (*P* = 0.77041) for HPN catheter days.

Abbreviations: CLABSI, central line–associated bloodstream infection; HPN, home parenteral nutrition; PICC, peripherally inserted central venous catheter; TCVC, tunneled central venous catheter.

* Statistics: two‐sample *z* test of proportions comparison of PICC vs TCVC.

Four patients had more than one CLABSI occurrence, each with a different CVC that were all counted as separate CLABSI events. One patient had four CLABSIs, each with a different organism, and another patient had three CLABSI events, one of which was multimicrobial. A third patient had two separate CLABSIs both with the same organism, one of which was multimicrobial. A fourth patient had two CLABSI episodes, each with a different organism. Of two patients who had depression and anxiety issues with suspicion of poor line care and partial noncompliance with HPN use, one had nine CVCs of which three were removed for CLABSI, and one had five CVCs of which four were removed for CLABSI.

Of the 23 HPN CLABSI cases, 14 occurred with ML catheters, most of which were DL, and nine occurred with SL catheters. HPN CLABSI rates were 1.33 for ML and 0.61 for SL catheters per 1000 total catheter days (*P* = 0.063) and 1.73 for ML and 1.22 for SL catheters per 1000 HPN catheter days (*P* = 0.542), with neither of the differences reaching statistical significance as depicted in Table 4. SL vs ML TCVC and PICC were also compared separately, as summarized in Table 5. CLABSI rates were 0.24 for SL TCVCs and 0.93 for ML TCVCs per 1000 total catheter days (*P* = 0.347) and 0.28 for SL TCVC and 1.16 for ML TCVC per 1000 HPN catheter days (0.322). CLABSI rates were 2.12 for SL PICC and 1.76 for ML PICC per 1000 total catheter days (*P* = 0.533) and 2.91 for SL PICC and 2.39 for ML PICC per 1000 HPN catheter days (*P* = 0.872)—not significantly different.

**Table 4:** HPN CLABSI rates—single lumen vs multiple lumen.

Infections	Overall (*n* = 23)	Single lumen (*n* = 9)	Multiple lumen (*n* = 14)	*P* value*
CLABSI per 1000 total catheter days	0.91	0.61	1.33	0.063
CLABSI per 1000 HPN catheter days	1.49	1.22	1.73	0.542

Abbreviations: CLABSI, central line–associated bloodstream Infection; HPN, home parenteral nutrition.

* Statistics: two‐sample *z* test of proportions.

**Table 5:** HPN CLABSI rates—SL vs ML/PICC and TCVC.

CLABSI rates (*n* = 23)	SL PICC (*n* = 8)	ML PICC (*n* = 9)	SL TCVC (*n* = 1)	ML TCVC (*n* = 5)
CLABSI per 1000 total catheter days	2.12	1.76	0.24	0.93
CLABSI per 1000 HPN catheter days	2.91	2.39	0.28	1.16

*Note*: SL PICC vs ML PICC per total catheter days: *P* = 0.533; per HPN catheter days: *P* = 0.872.

SL TCVC vs ML TCVC per total catheter days: *P* = 0.347; per HPN catheter days: *P* = 0.322.

Abbreviations: CLABSI, central line–associated bloodstream infection; HPN, home parenteral nutrition; ML, multiple lumen; PICC, peripherally inserted central venous catheter; SL, single lumen; TCVC, tunneled central venous catheter.

Of the 23 HPN CLABSI cases in our cohort, 52.2% of the causative organisms were gram‐positive bacteria, 26% gram negative bacteria, 4.3% both gram positive and gram negative, and 17.4% fungal infections (yeast). Of the total 23 CLABSIs, 4 (17.4%) were polymicrobial. *Staphylococcus* species were identified as the infecting organism in 30% of our CLABSI cases and found in 2 polymicrobial CLABSIs in our cohort. Table 6 lists microorganisms identified for the HPN CLABSI cases.

**Table 6:** Microorganisms identified for HPN CLABSI (*n* = 23).

Organism type	Microorganism
Gram positive (*n* = 12)	‐ *Staphylococcus* epidermidis (3 cases)
	‐ *Staphylococcus* carnosus, ssp carnosus
	‐ Kocuria kristinae
	‐ *Staphylococcus* aureus (2 cases)
	‐ Bacillus species, not anthracis, possible contaminant (CVC), Coagulase negative *Staphylococcus*, possible contaminant, and Viridans *streptococcus*, possible contaminant (peripheral)
	‐ *Staphylococcus* epidermidis/coagulase negative *Staphylococcus*
	‐ Group B *Streptococcus*
	‐ Enterococcus
	‐ Enterococcus casseliflavus
Gram negative (*n* = 6)	‐ Pseudomonas aeruginosa, Achromobacter, and Stenotrophomonas maltophilia
	‐ *Escherichia* coli
	‐ Enterobacter cloacae
	‐ Klebsiella pneumoniae
	‐ Klebsiella pneumoniae and *Escherichia* coli
	‐ Pseudomonas aeruginosa
Both gram positive and gram negative (*n* = 1)	‐ *Staphylococcus* hominis, and Klebsiella pneumoniae
Yeast (*n* = 4)	‐ *Candida* rugosa
	*‐ Candida* lusitaniae (2 cases)
	‐ *Candida* glabrata

Abbreviations: CLABSI, central line–associated bloodstream infection; CVC, central venous catheter; HPN, home parenteral nutrition.

Thirteen of the 23 CLABSI cases (nine with PICC and four with TCVC) qualified as estimated CRBSI based on our modified CRBSI definition. Estimated CRBSI rates were 0.51 per 1000 total catheter days overall, 1.01 for PICC, and 0.42 for TCVC (*P* = 0.131); and when calculated per 1000 HPN catheter days, CRBSI rate was 0.84 overall, with 1.38 for PICC and 0.51 for TCVC (*P* = 0.084).

Four infections were identified as non‐HPN CLABSI. Although there were no HPN CLABSIs with implanted ports, one port was removed for hospital CLABSI (*Escherichia* coli) 10 days after a hospital admission for pelvic surgery. One hospital CLABSI (*Candida* albicans) occurred with a TCVC 27 days after a hospital admission for a wound with copious drainage from an enterocutaneous fistula. A third non‐HPN CLABSI (Klebsiella pneumoniae) occurred in a patient with a TCVC 23 days after HPN was discontinued and 6 days after hospital discharge when the patient was no longer using PN. The fourth case occurred with a TCVC 10 days into a hospitalization that was originally classified as a urinary tract infection (UTI) by the medical team; however, it was determined to be a hospital CLABSI (Citrobacter freundii) after IP review because the bacterial count was not high enough to meet the NHSN surveillance definition for a UTI.

Alternate‐source infections were identified using NHSN criteria, as intra‐abdominal, GI, or pelvic abscess for six cases; urinary tract or urostomy (UTI) for three cases; and cardiovascular/endocarditis in two cases. One of the reported insertion‐site infections was with a PICC line with negative blood cultures from peripheral and CVC draw; and one was with a TCVC with purulence at the site, with no fever and no blood cultures drawn.

### Noninfectious complications

Twelve catheters were removed because of occlusion (four TCVC and eight PICC), 10 for malposition (five TCVC and five PICC); two for thrombosis (PICC), two for a leak (one TCVC and one PICC), and four for accidental dislodgement/removal (three TCVC and one PICC), of which two TCVC and one PICC belonged to one patient. One TCVC was removed because a patient with altered mental status, per registered nurse report, purposefully cut his TCVC with scissors during a hospitalization. There were more occlusions and thrombosis with PICC than with TCVCs, as shown in Table S3; however, the difference was not statistically significant. Occlusions occurred from 16 to 543 days of use, for an average time to occlusion of 179 days. The removal rates for noninfectious complications combined was 1.23 per total catheter days overall, with 1.91 for PICC, 1.46 for TCVC, and zero for ports. When calculated using HPN catheter days, the removal rate for noninfectious complications combined was 2.0 overall, with 2.61 for PICC, 1.78 for TCVC, and zero for ports. The differences in noninfectious complication rates between TCVC and PICC were not statistically significant per total catheter days (*P* = 0.453) and for HPN catheter days (*P* = 0.285). Seventeen catheters were in patients who died, none of which had CLABSI.

### Other clinical and demographic variables

As shown in Table 2, no significant differences were found in HPN CLABSI rates related to the main indications for PN, the presence of a fistula alone or ostomy alone, cancer diagnosis, gender, or race. HPN CLABSI rates were significantly higher for central lines in patients who had both a fistula and an ostomy as compared with those who had neither, per HPN days (*P* = 0.023) and total catheter days (*P* = 0.029). CLABSI rate per HPN catheter days was higher for central lines in patients with a fistula, though it did not reach statistical significance (*P* = 0.054). We found a lower incidence of HPN CLABSI—0.612 per 1000 HPN catheter days—for CVCs in patients who did not have home nursing, as compared with 1.589 per 1000 HPN catheter days for those who had home nursing, yet the difference was not significant (*P* = 0.332). CLABSI rates were lowest for patients with commercial insurance compared with Medicare, Medicaid, or no coverage. For the total 141 CVCs, the average age of patients at the time of line placement was 51.4 years (SD 14.1 years).

The average patient age at the time of CVC placement was 55.5 years for CVCs later found with CLABSI and 50.6 years for CVCs without CLABSI with no significant differences (*P* = 0.1039) between the two age groups. However, HPN CLABSI rates were significantly lower for patients younger than 43 years as compared with those of aged ≥43 years per HPN days (*P* = 0.016) and per 1000 total catheter days (*P* = 0.012).

## DISCUSSION

Comparisons of bloodstream infections in HPN patients is complicated because the definitions vary among different researchers and methods are sometimes not clearly defined, with most using the term CRBSI and some using CLABSI.^1^, ^2^, ^3^ In the 17‐study meta‐analysis by Hon et al, only 7 had strict protocols for the diagnosis of CRBSI^5^; and in the 53‐study meta‐analysis by Reitzel et al, studies were graded on a 4‐level scale based on the stringency of criteria used to determine CLABSI or CRBSI.^1^ Tribler et al illustrated the difference between clinically reported CRBSI vs strict CDC ESPEN CRBSI diagnosis. CRBSI rates per 1000 catheter days for the Copenhagen intestinal failure database were 1.95, and when reevaluated by CDC microbiologic criteria, the rates were much lower at 0.92.^2^ Similarly, we originally calculated a clinically based CLABSI rate of 1.74 per 1000 HPN catheter days overall, 3.07 for PICC, and 0.89 for TCVC,^21^ and after application of NHSN criteria, our CLABSI rates were recalculated as in this report, 1.49 per 1000 HPN catheter days overall, 2.61 for PICC, and 0.76 for TCVC.

Clinical observations in daily practice cannot capture CRBSI adequately because the required level of testing is not consistently performed; thus, overall rates can appear lower simply because of a lack of testing. ESPEN and the Infectious Disease Society of America have published stringent quantitative and differential time to positivity microbiologic criteria for the diagnosis of CRBSI.^22^, ^23^ Tribler et al expanded on the problem of accuracy in CRBSI determination, noting that paired culture positivity with the same microorganism species in CVC and peripheral vein blood cultures was supported in an average of ~40% of their CRBSI episodes, and of the catheters that were removed, 13% were verified by a positive catheter tip.^2^ In comparison, our CRBSI estimates were based on paired culture positivity in 13 of the 23 (>56.5%) of our CLABSI cases, with 10 cases (43%) further confirmed by a positive catheter tip culture.

Our findings of higher bloodstream infection rates with PICC lines than with TCVC did not align with the majority of the literature reviewed.^1^, ^5^, ^6^ Table 7 summarizes infection rates and details from selected studies. Part of the difference between our findings and those of others may be because of differences in the number of oncologic patients in the populations observed, as cancer patients are at increased risk for CLABSI.^27^ Santarpia et al reported no differences related to catheter type, and 74% of their study population were oncologic patients.^24^ In contrast, only 47% of our patients had a cancer diagnosis. Botella‐Carretero et al reported no confirmed infections with PICC lines, very high CRBSI rates for TCVC and ports, and 82% of patients with oncologic diagnoses.^25^ Santacruz et al had fewer confirmed CRBSI episodes with PICCs than with ports and 83% of patients with malignancy.^7^ Vashi et al found no significant differences in CRBSI rates between PICC, TCVC, and ports in their group of patients with cancer receiving HPN.^26^ Another reason our results may differ from others is the fact that our patients had blood drawn from their CVCs for routine laboratory tests. Buchman et al found that the practice of drawing blood from the CVC was a significant risk factor for infection.^15^

**Table 7:** Infection data comparison from selected observational studies.

Author and type of study	Infection definition	Infection rate per 1000 catheter days				Significant findings^a^	Patients with cancer	Number of patients (mean age ± SD, in years)	Number and type of catheters
		Overall	PICC	TCVC	Port				
Santacruz et al.^7^ Prospective	CRBSI	n/a	0.15	0.72	2.02	Lower rate with PICC than port, no difference between PICC and TCVC, higher rate with ML than SL.	82.7%	151 (58 ± 13)	116 PICC 18 TCVC 36 Port
Toure et al.^9^ Prospective	CRBSI	n/a	1.05	1.87	n/a	Lower rate with PICC vs TCVC.	16.3% (active cancer)	196 (55.6 ± 16.5)	71 PICC 133 TCVC 0 Port
Ross et al.^13^ Prospective	CLABSI	0.87 total^b^ 1.17 (children) 0.35 (adults)	0.41	0.51	0.66	Higher rate with ports than with PICC, higher rate with DL (0.57) than with SL (0.38) catheters.	18.7% had GI cancer	138 children age <18 y, 908 adults age ≥18 y	449 PICC 492 TCVC 93 Port 11 Other
Xue et al.^14^ Retrospective QI	CLABSI	0.89	Not reported.			TCVC and ports were higher predictors of CLABSI than PICC.	25.4%	114 (54 ± 16)	83 PICC 27 TCVC 4 Port
Santarpia et al.^24^ Retrospective	CRBSI	1.74	Not reported.			Higher infection risk with previous catheterization and enterocutaneous stoma.	73.8%	172 (62 ± 13.5)	27 PICC 37 TCVC 125 Port 49 Other^c^
Buchman et al.^15^ Retrospective	CRBSI	0.35	n/a	0.32	0.66	Higher risk with ports, ML catheters, and obtaining blood from CVC.	Unknown	125 Median age 58 y – women 49 y – men	0 PICC 268 TCVC 63 Ports
Cotogni et al.^19^ Prospective cohort	CRBSI	0.29	0.08	0.57	0.21	PICC and port lower rate than TCVC.	100%	761 Median age 64	401 PICC 118 TCVC 198 Port 137 Other^c^
Botella‐Carretero et al.^25^ Prospective	CRBSI	n/a	0	20	44	Lower rate with PICCs vs ports.	81.9%	72 (58.5 ± 12.9)	48 PICC 10 TCVC 21 Port
Vashi et al.^26^ Retrospective cohort	CRBSI	0.54	0.61	0.93	0.47	No differences among catheter types.	100%	335 (53.7 ± 9.3)	191 PICC 11 TCVC 206 Port
Zhao et al.^10^ Retrospective	IRR	n/a	1.78	1.00		Higher rate with PICC than with non‐PICC.	4.9% cancer as primary indication for PN	101 (51 ± 13)	123 PICC 60 TCVC 12 Port 1 vascath
Christensen et al.^12^ Retrospective cohort	CRBSI	n/a	1.63	0.56	n/a	Higher rate with PICC than with TCVC, and time to CRBSI shorter for PICC. Higher rate for TCVCs in patients with home nurses.	8.8% of catheters in cancer patients	136 (64 ± 13) with TCVC; (65 ± 11) with PICC	126 PICC 169 TCVC
Bech et al.^11^ Retrospective, cohort	CRBSI	n/a	1.43	0.95	n/a		6.6% (active cancer)	136 (60.7 ± 17.3)	

Abbreviations: CLABSI, central line–associated bloodstream infection; CRBSI, catheter‐related bloodstream infection; DL, double lumen; GI, gastrointestinal; IRR, incidence rate ratio; ML, multiple lumen; PICC, peripherally inserted central catheter; port, implanted port; QI, quality improvement; SL, single lumen; TCVC, tunneled central venous catheter; y, years.

^a^ Key information to compare with this project.

^b^ Rates based on HPN days.

^c^ Nontunneled catheter.

As summarized in Table 7, only a few studies in the literature show a higher infection rate with PICC lines in patients receiving HPN. Zhao et al reported a significantly higher incidence rate ratio for bloodstream infections with PICC as compared with non‐PICC, and similar to our population, none of the patients used ethanol lock.^10^ Like our methods, Zhao used the concept of HPN catheter days as well as total catheter days in calculation of infection rates.^10^ Two other reports, both analyzing the same cohort of patients in Denmark, reported significantly lower incidence of CRBSI with TCVC than with PICC.^11^, ^12^

We found that CVCs used by patients with no home nursing had a lower yet not statistically significant difference in incidence of CLABSI than those who had weekly home nursing visits, and patients younger than 43 years had significantly lower CLABSI incidence (*P* = 0.012). Similarly, Brandt et al reported a higher hazard ratio for CRBSI for patients with HPN administered by home nurses than those who did not have home nurses and a significantly lower CRBSI hazard ratio for the age group of 40–50 years as compared with older patients in their cohort.^28^ Bech et al also found that TCVCs managed by a home care nurse had a significantly higher CRBSI rate than TCVCs not managed by nursing.^11^ In contrast, Bond et al reported the lowest CRBSI rate for their patients who had trained home nurses.^29^

Our overall CLABSI rate of 0.91 per 1000 total catheter days is within the lower range of 0–11.89 per 1000 catheter days found in prior published studies of HPN. However, our CLABSI rate for PICC lines alone is 1.91, higher than the range of 0–1.63 rate reported in the literature.^1^ Our TCVC CLABSI rate of 0.63 is within range and lower than some reports of 0.32–3.3.^1^, ^3^, ^30^ Our CRBSI estimates of 1.01 per 1000 catheter days for PICC and 0.42 for TCVC are within previously reported ranges of 0–1.96 for PICC and 0.19 to 20 for TCVC.^1^, ^5^, ^30^, ^31^, ^32^

The majority of HPN CLABSIs identified (56.5%) in this project had positive cultures for gram‐positive bacteria. This is similar to CLABSI in acute care hospitals, in which 60% of events are caused by gram‐positive bacteria.^33^ Our HPN CLABSI findings of 52.2% gram‐positive bacteria, 26.1%, gram‐negative bacteria, 4.3% both gram positive and gram negative, and 17.4% *Candida* species are similar to the meta‐analysis by Reitzel et al, who reported that 53% of HPN CLABSI were from gram‐positive and 26% by gram‐negative organisms and 12% were from *Candida* yeast.^1^ Our finding of *staphylococcus* species as the infecting organism in 30% of our HPN CLABSI cases is also similar to their finding of 34% of the causative organisms as *staphylococcus* species.^1^

This project was the initial measurement phase of QI to determine if differences exist in infection rates or other complications between the three types of CVCs used in our HPN population. Next steps in the QI project would include sharing the outcomes with providers who order HPN, followed by implementation of practice changes, such as decreasing the frequency of PICC line use to potentially reduce infection risk. Future data collection to reevaluate HPN CLABSI rates after practice changes are implemented would be necessary to complete a QI cycle.

### Strengths

Potential CLABSI events were verified by application of CDC‐NHSN criteria by an IP expert, which confirmed the validity of our results. We also analyzed each infection episode to categorize by a more stringent definition, an estimate of CRBSI. In addition to the standard total catheter days, we also calculated HPN catheter days to provide a specific look at the time patients were receiving our service using HPN. Focusing on HPN use with a single infusion provider is logical for quality measurement and because PN has been found to be an independent risk factor for CVC infections.^1^ Our prospective data collection minimized errors from misused terminology and missing information. Chart review revealed some clinicians misused the terms “PICC” and “port” for tunneled catheters. The lead author verified correct CVC types through interventional radiology CVC insertion notes and identified unrecorded hospital admissions and new catheter placements that might be missed retrospectively.

### Limitations

This single‐center observation is relatively small compared with studies that dealt with higher numbers of patients. We relied on tests deemed appropriate for individual patients. Comparison of our results with published studies is limited because of differences in the definitions used for HPN CLABSI and CRBSI.^1^, ^2^ We did not keep track of any other IV fluids, such as IV hydration, pain control, or IV antibiotic use. A small number of patients in our cohort used 70% ethanol‐impregnated caps instead of standard caps to cover CVC hubs when not in use, and at least 1 patient used a Biopatch under the CVC dressing, neither of which was standard practice, and information on use of these items was not tracked. Ethanol‐impregnated caps and Biopatch are thought to reduce infection risk, but more study is needed.^34^, ^35^ Our data collection spanned the time of the worldwide COVID‐19 outbreak; thus, enhanced infection control measures, social distancing, and staffing shortages could have affected infection rates.

## CONCLUSIONS

Observations from this initial phase of a QI project revealed a significantly lower HPN CLABSI risk with TCVCs as compared with PICC lines, and no HPN CLABSI occurred with implanted ports for patients using HPN at our health system. HPN CLABSI rates were significantly lower for patients younger than 43 years, and significantly higher for patients with both an enterocutaneous fistula and an ostomy. HPN CLABSI rates were higher for ML as compared with SL catheters, and there were more noninfectious complications with PICCs than with TCVCs, yet the differences did not reach statistical significance. Our overall HPN CLABSI rates are within the range reported in the literature, yet our findings differ from most studies, which found lower rates of infection with PICC as compared with TCVC and ports. Our findings will be used to help guide decisions regarding CVC choice for our patients who will recieve HPN. Future data collection is recommended to reevaluate HPN CLABSI rates after practice changes are implemented. Also, future studies are needed that use consistent definitions for CLABSI or CRBSI and control for variables, such as CVC care, nursing, and blood draw protocols.

## AUTHOR CONTRIBUTIONS

Theresa A. Fessler conceived and designed the research, contributed to the acquisition, analysis and interpretation of the data, and drafted and critically revised the manuscript; Mary B. Crandall contributed to the research design, the analysis and interpretation of data, and critically revised the manuscript; and Sarah E. Schumacher and David N. Martin contributed to the analysis and interpretation, and critically revised the manuscript. All authors have read the manuscript, gave final approval, and agreed to be fully accountable for ensuring the integrity and accuracy of the work.

## CONFLICT OF INTEREST STATEMENT

None declared.

## Supporting information

Supplementary Table S1 ‐ Copy.

Supplementary Table S2 ‐ Copy.

Supplementary Table S 3 ‐ Copy.
