# Supplementary material for: Central line–associated bloodstream infections and complications in adult home parenteral nutrition: Observations from a quality improvement initiative
Source: Nutr Clin Pract. 2025 Jun 29;40(6):1612–28. doi: 10.1002/ncp.11338 (PMC12590316; doi:10.1002/ncp.11338)
Supplement: Supplementary file 1 — Supplementary Table S1 ‐ Copy. [file NCP-40-1612-s001.docx]

**Table S1.**  Types of CVCs and CLABSI events

| Types of CVCs | CVCs  (n =141) | No HPN CLABSI  (n = 118) | HPN CLABSI  (n = 23) |
| --- | --- | --- | --- |
| PICC  TCVC  Port | 89  38  14 | 72  32  14 | 17  6  0 |
| Total ML  Total SL  Unknown # of lumens | 80  60  1 | 66  51  1 | 14  9  0 |
| PICC SL  PICC DL  PICC TL | 35  52  2 | 27  44  1 | 8  8  1 |
| TCVC SL  TCVC DL  TCVC TL | 13  24  1 | 12  19  1 | 1  5  0 |
| Port SL  Port DL  Port unknown lumens | 12  1  1 | 12  1  1 | 0  0  0 |

CLABSI, central line associated bloodstream infection; CVCs, central venous catheters; DL, double lumen; HPN, home parenteral nutrition; ML, multiple lumen; PICC, peripherally inserted central venous catheter; Port, Implanted port; SL, single lumen; TCVC, tunneled central venous catheter; TL, triple lumen.
