# Supplementary material for: Central line–associated bloodstream infections and complications in adult home parenteral nutrition: Observations from a quality improvement initiative
Source: Nutr Clin Pract. 2025 Jun 29;40(6):1612–28. doi: 10.1002/ncp.11338 (PMC12590316; doi:10.1002/ncp.11338)
Supplement: Supplementary file 2 — Supplementary Table S2 ‐ Copy. [file NCP-40-1612-s003.docx]

**Table S2**: Catheter days

| Type of CVC | Total Catheter Days (n = 25,273) | HPN Catheter Days (n = 15,474) |
| --- | --- | --- |
| PICC  TCVC  Port | 8879  9577  6817 | 6509  7868  1097 |
| SL  ML  Unknown | 14702  10550  21 | 7363  8090  21 |

CVC, central venous catheter; HPN, home parenteral nutrition; ML, multi lumen; PICC, peripherally inserted central venous catheter; Port, implanted port; SL, single-lumen; TCVC, tunneled central venous catheter; Unknown, unknown lumens
