# Supplementary material for: Central line–associated bloodstream infections and complications in adult home parenteral nutrition: Observations from a quality improvement initiative
Source: Nutr Clin Pract. 2025 Jun 29;40(6):1612–28. doi: 10.1002/ncp.11338 (PMC12590316; doi:10.1002/ncp.11338)
Supplement: Supplementary file 3 — Supplementary Table S 3 ‐ Copy. [file NCP-40-1612-s002.docx]

**Table S 3:** Non-Infectious Complications (n=31)

| CVC Removal reason | Total | PICC | TCVC | Port |
| --- | --- | --- | --- | --- |
| Thrombosis | 2 | 2 | 0 | 0 |
| Malposition | 10 | 5 | 5 | 0 |
| Occlusion | 12 | 8 | 4 | 0 |
| Leak | 2 | 1 | 1 | 0 |
| Accidental Dislodgement | 4 | 1 | 3 | 0 |
| Other ^a^ | 1 | 0 | 1 | 0 |
| Total | 31 | 17 | 14 | 0 |
| Non-Infectious Complication Rates  Per 1000 Total Catheter Days* | 1.23 | 1.91 | 1.46 | 0 |
| Non-Infectious Complication Rates  Per 1000 HPN Catheter Days ** | 2.00 | 2.61 | 1.78 | 0 |

CVC, central venous catheter; PICC, peripherally inserted central venous catheter; Port, implanted port; TCVC, tunneled central venous catheter.

^a^ Other- Patient with altered mental status cut catheter with scissors

*p = 0.453, **p = 0.285, Statistical comparisons between PICC and TCVC using 2 sample z test of proportions.
